# Conformal sublobar electroresection with volume optimization achieves greater parenchymal preservation than stapler in wedge resection: a volumetric analysis

**DOI:** 10.3389/fonc.2025.1657405

**Published:** 2025-10-27

**Authors:** Xining Zhang, JingWei Liu, ShiJie Zhang, Jian Li

**Affiliations:** Peking University First Hospital, Beijing, China

**Keywords:** small pulmonary nodule, NSCLC, minimally invasive surgery, wedge resection, diathermy

## Abstract

**Background:**

Wedge resection is widely applied for small pulmonary nodules, yet stapler-based approaches may result in unnecessary parenchymal sacrifice. The CONSERVO (Conformal Sublobar ElectroResection with Volume Optimization) technique is an electrosurgical, shape-respecting method designed to maximize parenchymal preservation while maintaining oncologic and procedural safety.

**Method:**

We retrospectively included 104 patients who underwent stapler-based or CONSERVO wedge resection for solitary pulmonary nodules at our center between 2023 and 2024. Propensity score matching and multivariable linear regression were conducted. Key outcomes included lobe to lung volume ratio loss, operative time, incision length, intraoperative bleeding, drainage volume, and complication rate.

**Result:**

Baseline characteristics were well-balanced between groups. The CONSERVO group demonstrated superior parenchymal preservation, with significantly lower lobe-to-lung volume ratio loss (4.66% [IQR 1.68%-7.02%] vs. 11.17% [IQR 8.91%-14.48%], p<0.001) and lobe volume loss (149855.5 ± 221949.3mm^3^ vs. 220374.6 ± 189597.7mm^3^, p<0.001). Multivariable regression analysis confirmed the parenchyma saving effect of the CONSERVO technique (Coef. = -0.133, 95% CI: -0.182 to -0.084, p < 0.001). Additionally, the CONSERVO group was associated with a shorter incision length (23mm [IQR 20-25mm] vs. 35mm [IQR 30-40mm], p<0.001). The operative time was longer in the CONSERVO group (133min [IQR 103-170min] vs. 62min [IQR51-104min], p<0.001). No significant differences were observed in thoracic drainage duration, postoperative drainage volume, or pathological outcomes.

**Conclusion:**

The CONSERVO approach achieves meaningful parenchymal preservation and offers additional advantages such as smaller incision size while maintaining oncologic and procedural safety. It may be particularly suited for patients with limited pulmonary reserve or those requiring multiple resections, as well as nodules located close to hilar structures.

## Introduction

Wedge resection remains a cornerstone in the surgical management of small pulmonary nodules, especially in the era of widespread CT-based lung cancer screening (1, 2). The rising detection of small, non-palpable, and often indeterminate nodules has increased demand for precise, limited parenchymal resections that achieve oncologic adequacy (3) while preserving maximal lung function (4, 5). Traditionally, such resections are performed using stapling devices, which offer speed and simplicity but may unnecessarily remove healthy lung tissue and distort the geometry of the remaining lobe. As thoracic surgery evolves toward increasingly function-oriented paradigms, more refinement of sublobar techniques to enhance anatomical preservation has become a critical need.

Recent studies have highlighted the limitations and pitfalls of conventional stapler-based resections in parenchymal-sparing surgery (6). Although effective in achieving negative margins, stapler resections create rigid linear planes that may not conform to the natural contour of the lung parenchyma, particularly when targeting deep or irregularly shaped nodules. This can result in disproportionate loss of functional tissue—an issue of increasing concern in patients with borderline pulmonary function or those with multiple nodules requiring repeated resections. One solution is to use radial, instead of linear loads, to obtain a more conformal margin (7). However, the radial load is still fixed in angle and width, thus cannot fit the desired contour of resection ubiquitously.

To address this gap, we developed the CONSERVO approach—Conformal Sublobar ElectroResection with Volume Optimization—an electrocautery-based, anatomy-respecting approach to wedge resection. This approach emphasizes conformality to lung anatomy, selective parenchymal dissection, and careful volume optimization, aiming to minimize tissue loss while maintaining procedural safety and oncologic soundness. In contrast to stapler resection, the CONSERVO approach allows the surgeon to tailor the resection path more precisely to the geometry of the nodule and surrounding lung, potentially enhancing functional preservation without compromising outcomes. In suitable situations, lobulectomy, i.e., resection of the nodule harboring lobules, was achievable to maximize the conservation of functional parenchyma in this study.

The present study retrospectively compares the CONSERVO approach to conventional stapler-based wedge resection in patients undergoing surgery for solitary pulmonary nodules. We hypothesized that the CONSERVO approach would result in significantly reduced parenchymal volume loss while maintaining comparable safety, pathology, and perioperative outcomes. This investigation also explores secondary benefits, such as reduced incision size and procedural bleeding, as well as the potential applicability of lobulectomy, i.e., lobule-based anatomical resection —under the framework of the CONSERVO approach.

## Methods

### Study design and patient selection

This was a retrospective, single-center cohort study conducted at our center between 2023 and 2024. The study included consecutive patients who underwent wedge resection for a solitary pulmonary nodule using either a conventional stapler-based approach or the CONSERVO technique. Inclusion criteria were (1): radiologically or clinically suspicious solitary pulmonary nodule ≤ 2 cm (2); peripheral location deemed appropriate for wedge resection; and (3) availability of complete preoperative and 6-month postoperative chest CT scans. Exclusion criteria included (1): multifocal disease (2); history of prior ipsilateral lung resection; or (3) conversion to lobectomy or segmentectomy intraoperatively. Our institution’s ethical board approved this study (Appro. ID 2025-0574). Informed consent was obtained from each patient.

### Surgical techniques

All procedures were performed under general anesthesia via uniportal thoracoscopic access. The choice of resection method—stapler-based or the CONSERVO technique—was based on surgeon preference, patient anatomy, and instrument availability. Six experienced thoracic surgeons (JL, SJZ, ZQL, JWL, HZ, and XN) conducted all the resections.

### Conventional stapler-based resection

Stapler-based wedge resection was performed using endoscopic linear staplers to encircle and excise the target lesion with a visually estimated margin of at least 1 cm. The margin was assessed intraoperatively through gross inspection and a frozen section. Additional stapler-based resection would be performed if a satisfactory margin was not achieved. A thoracic drainage tube of 16 or 20 Fr was placed through the incision before closure.

### CONSERVO technique

**Figure 1:**
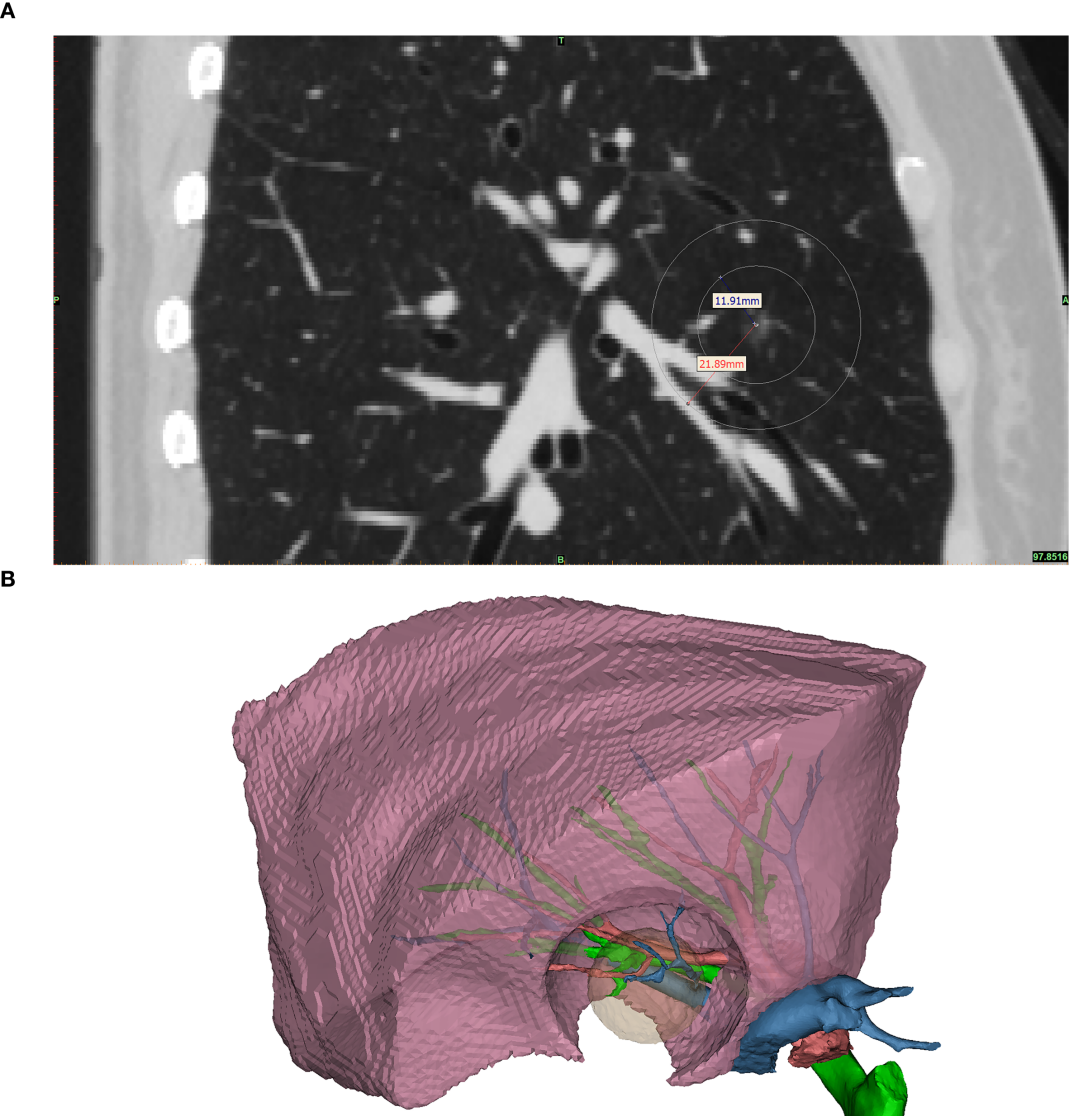
The illustrated middle lobe resection range. This figure shows the required resection range and the area of parenchyma that would be affected by the staplers. **(A)** shows the sagittal plane of the same patient’s preoperative CT scan. The nodule was located at the center of the concentric circles; the inner circle marked the required range of a satisfactory resection, and the outer circle marked the parenchyma that would be affected by the 10mm width staplers. Noted that for this patient, a right middle lobectomy was warranted if stapler-based resection was to be applied, as the stapler would inevitably affect the major bronchi, pulmonary arteries, and veins of the middle lobe. **(B)** 3D reconstructed model of the middle lobe. The pink object is the reconstructed right middle lobe. The green, red, and blue branching objects are the reconstructed bronchus, pulmonary artery, and vein, respectively. The translucent yellow object is the required resection range, and the hole-like contour in the right middle lobe is the parenchyma that would be affected by the staplers. Noted that a resection by staplers would affect the key structures of the right middle lobe, causing a significant amount of loss of healthy parenchyma.

The CONSERVO technique employs a diathermy-based approach to perform wedge resections that conform to the anatomical contour of the lung parenchyma. This technique is designed to minimize unnecessary tissue loss while preserving functional architecture. The major advantage of the diathermy pen, when compared to the staplers, is the millimeter-level accuracy and the ability to shape a non-rigid, thin resectional plane, which is frequently required to preserve adjacent bronchi and pulmonary vasculature (**Figure 1A**). The required range of resection and the parenchyma that would be affected by the staplers was better illustrated in the 3D reconstructed model (**Figure 1B**).

**Figure 2:**
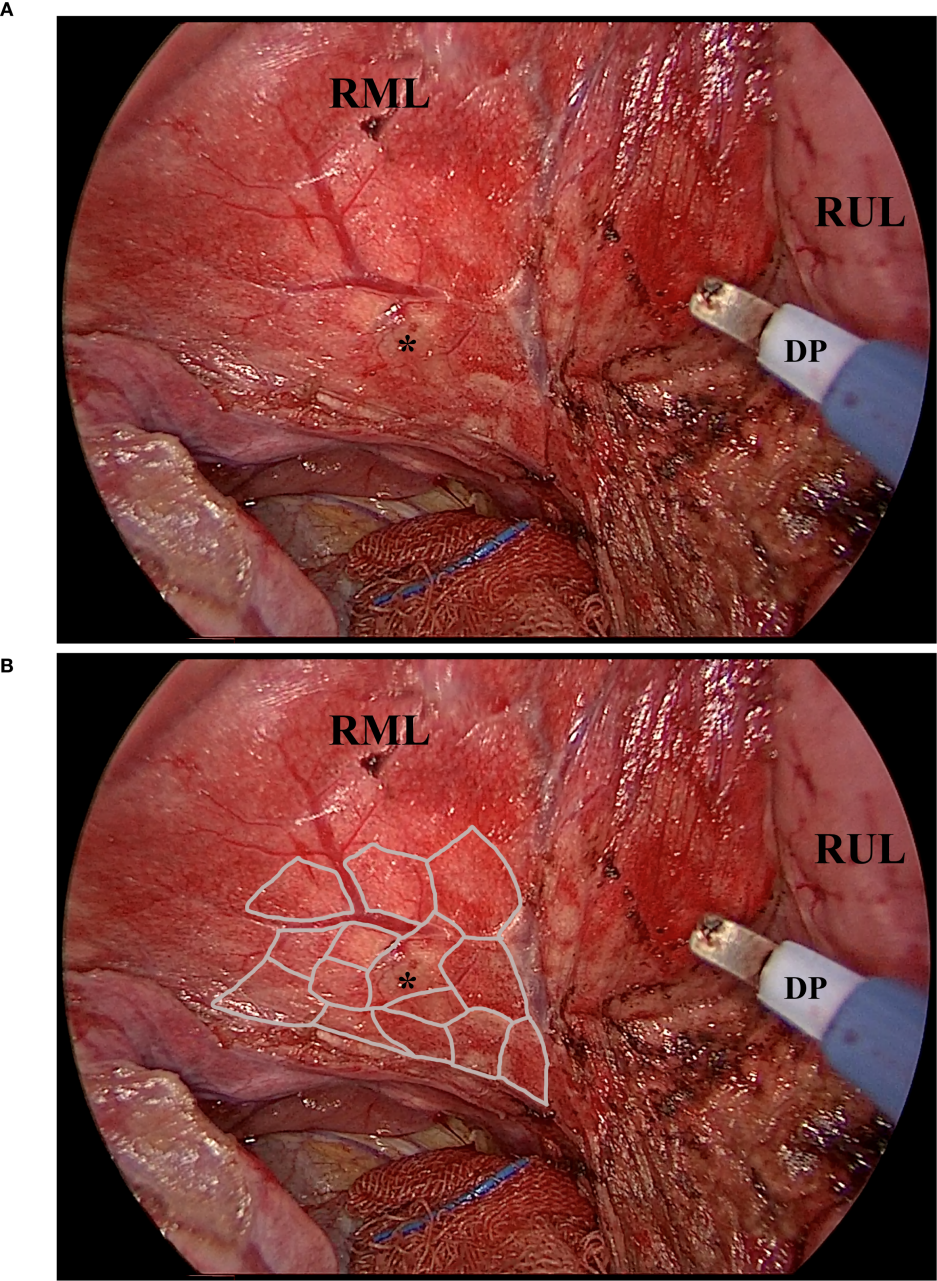
The fully expanded parenchyma and the lobules. This picture, captured intraoperatively, shows the expanded parenchyma, the lobules, and the working diathermy pen. Noted that the diathermy pen was adjusted to an angle of about 40 degrees to facilitate instrument cooperation. **(A)**, the exposed parenchyma of the right middle lobe after the right horizontal fissure was divided by the diathermy pen. The sub-fissure pulmonary vein was visible, as were the nodule-bearing lobules. **(B)**, the illustrated margin of the lobules. Noted that the expanded parenchyma, plus the thin edge of the diathermy pen, made the lobule-based resection achievable. Asterisk, the nodule that is to be resected. DP, the diathermy pen. RML, the right middle lobe. RUL, the right upper lobe.

Two core strategies were applied to improve margin control and geometric precision. First, the resection was performed using a fine electrocautery pen rather than a linear stapler, providing enhanced maneuverability and contour adherence. Second, a “map zooming” concept was used: the target lung is expanded via bilateral ventilation (tidal volume 5 mL/kg), allowing for clearer visualization of lobular boundaries (**Figure 2A**). In cases with prominent interlobular septa, this expansion enables even lobule-oriented resections (**Figure 2B**).

**Figure 3:**
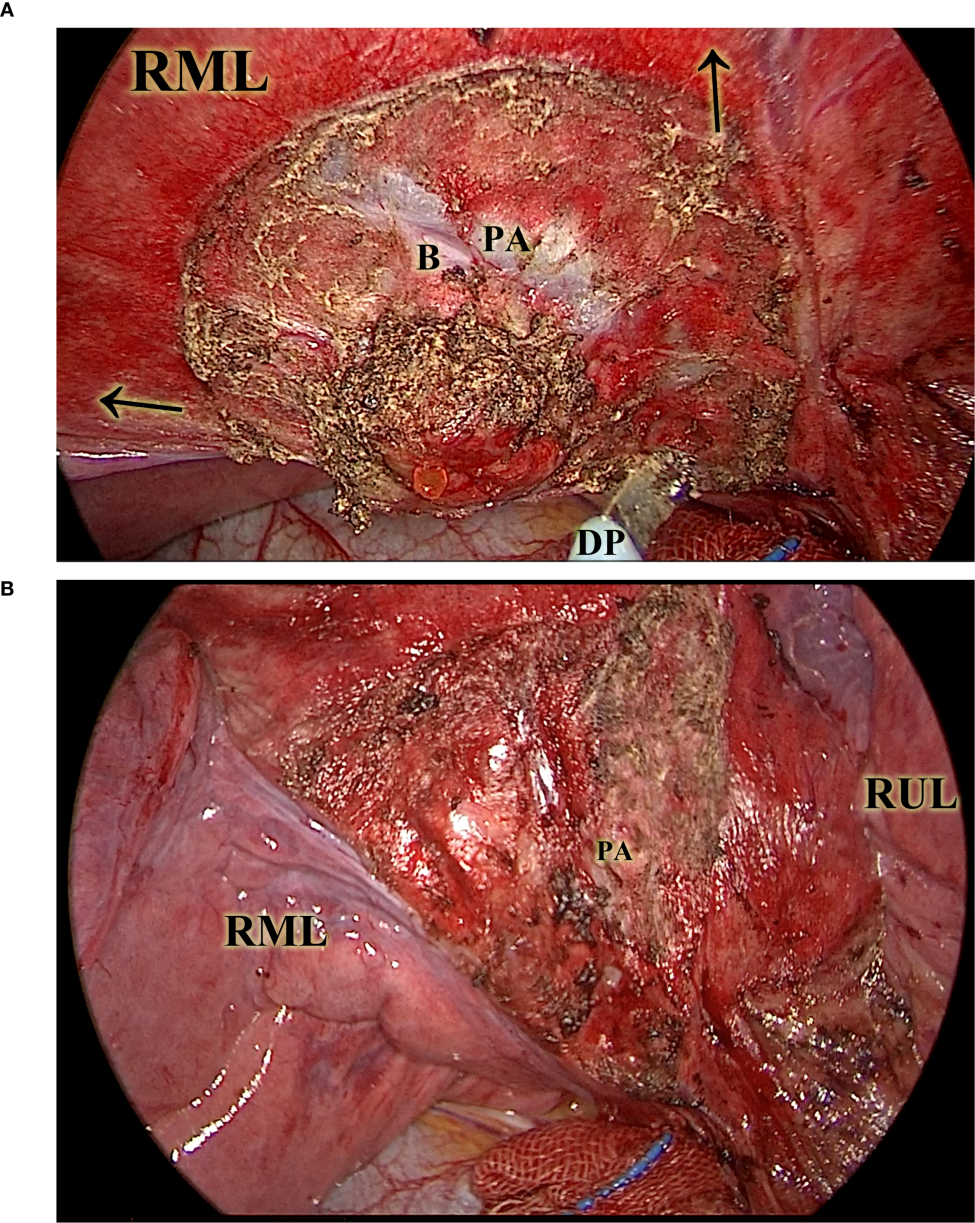
The diathermy-based resection. This picture illustrates the resection in a CONSERVO resection. The ventilated lung greatly enhanced the visibility of lobule-based structures. **(A)**, the diathermy pen, under the vectorial traction (illustrated by the arrows) of the suction tip and the thoracoscopic forceps (not included in this picture), was able to follow the planned lobule-based resection on ventilated parenchyma. **(B)** shows the right middle lobe after diathermy, with the key structures, such as the pulmonary artery of the right middle lobe, protected and preserved. B, the right middle lobe bronchus. DP, the diathermy pen. PA, the right middle pulmonary artery. RML, the right middle lobe. RUL, the right upper lobe.

Nodules were identified via visual inspection, palpation, or preoperative CT-guided localization, as appropriate. Once the targeted lobe was fully ventilated and turned pink, the intended resection area was marked on the visceral pleura using electrocautery. Parenchymal dissection was performed using a combination of suction and forceps, applying dynamic bidirectional traction (**Figure 3A**). The resection followed the anatomical cone shape of the parenchyma, which typically consists of several lobules, necessitating frequent intraoperative inspection to maintain orientation. Traction vectors were adjusted during the dissection to achieve circumferential margins. During the procedure, small pulmonary veins along the septa were safely cauterized without ligation. The dominant bronchus and artery—typically encountered at the apex of the resectional cone—were exposed and divided at approximately 10 mm beyond the deep margin of the nodule (**Figure 3B**).

**Figure 4:**
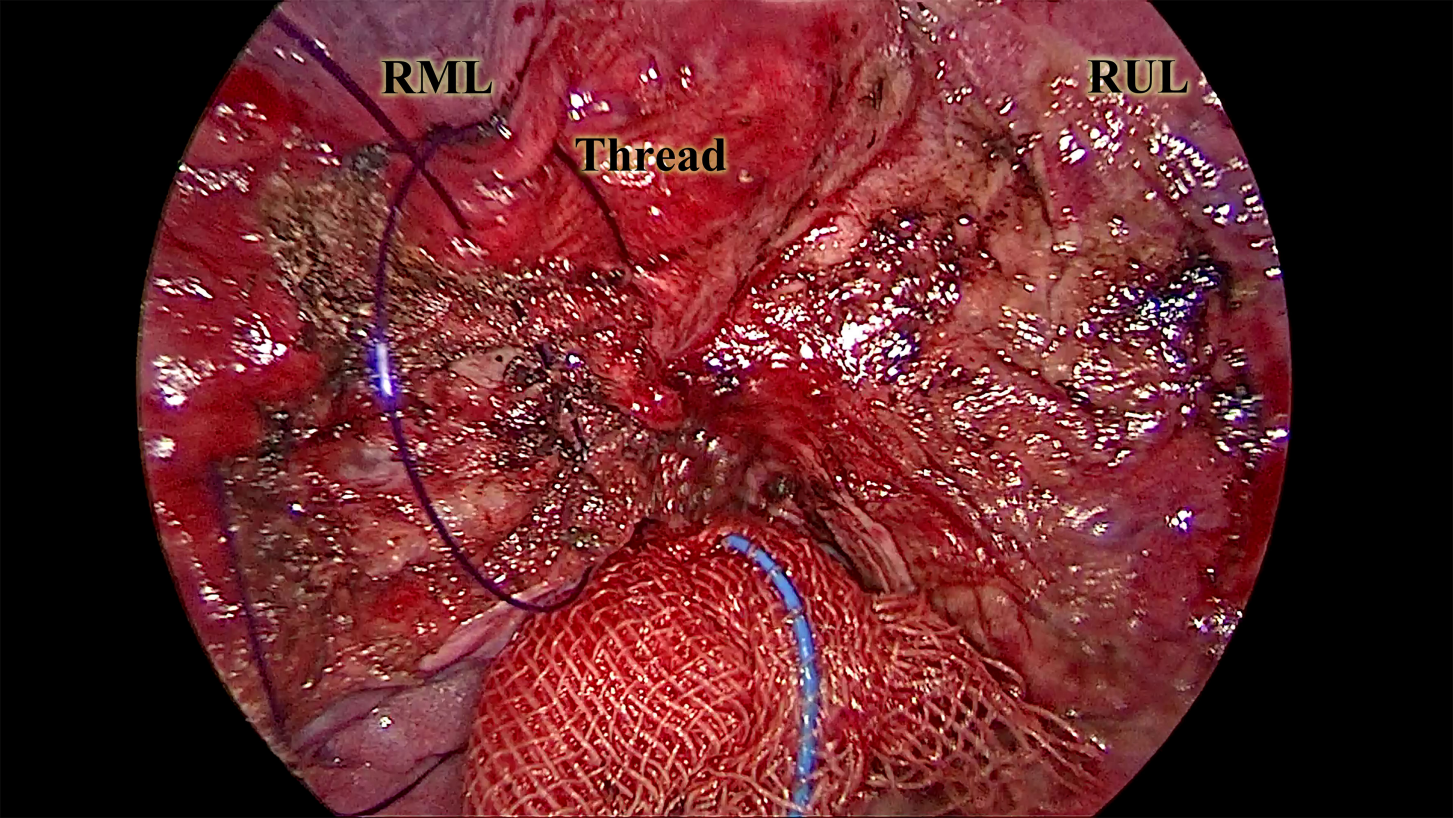
The full-thickness suture of the exposed parenchyma. This picture, captured from the continuous suture of the exposed parenchyma, illustrates a critical part of the CONSERVO technique. The purple 3–0 PDSII thread was sutured through the adjacent pleura and the exposed parenchyma. The depth of the suture was maintained at 2–3 millimeters to prevent injury to the underlying bronchus and vasculature. This suturing technique prevents alveolar-pleural fistula, enhances parenchyma preservation, and minimizes postoperative adhesion. RML, the right middle lobe. RUL, the right upper lobe.

**Figure 5:**
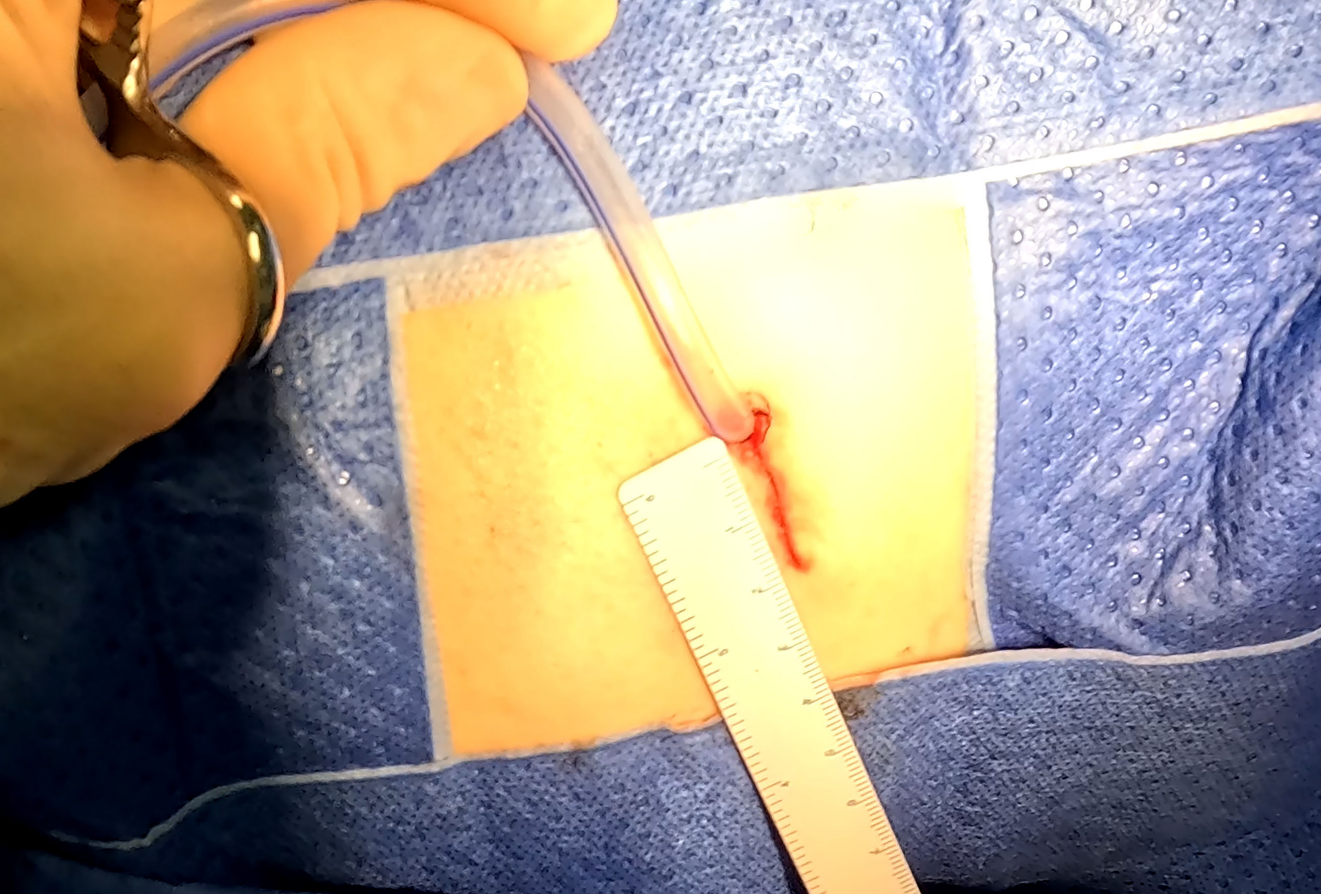
The sutured incision of a CONSERVO resection. This picture shows a typical incision of the CONSERVO resection. Without the requirement of staplers, a 2-centimeter incision was enough for dissection and suturing.

Following resection, a water-seal test under bilateral ventilation was performed to identify air leaks. Major leaks were repaired using continuous 5–0 PDS*II (Ethicon Inc.) sutures. The remaining parenchymal defect was closed with continuous, full-thickness sutures using the same material in the deflated state of the lung (**Figure 4**). Full-thickness closure was essential to prevent postoperative intraparenchymal cavity formation and pleural adhesion formation. Notably, without the need to allow the stapler to pass through the incision, our technique enables complex instrument interaction under the setting of a 2-centimeter-long incision (**Figure 5**). The subsequent steps mirrored standard wedge resection protocol.

### Imaging and volume measurement

All patients underwent preoperative and 6-month postoperative high-resolution chest CT scans (120kVp, 35mA, 0.625 slice thickness) using SOMATOM Definition AS (Siemens Medical Solutions USA, Inc. ^©^2025, Pennsylvania, US). Lobe and total lung volumes were measured using a free-trial version of Mimics software (Materialise NV, Leuven, Belgium). All the 3D model was constructed using the automated “Segment Lung and Lobes” function provided by the software. The volumetric information of the reconstructed 3D models of the respective lobe and lung of each patient was then collected and analyzed. The volumetric reconstructions and metrics collected were reviewed by two independent thoracic surgeons (JL and SJZ). As the volume of the lung and its lobes can be influenced by the extent of inhalation and chronic pulmonary diseases, the primary metric of interest was the lobe volume loss normalized to the ipsilateral lung volume, expressed as a percentage of the ipsilateral lung volume, i.e., the loss of lobe/lung ratio. Other metrics, such as lung and lobe volume loss and percentage of volume loss, were also calculated and recorded.

### Postoperative outcomes and pathological assessment

Perioperative metrics included operative time, incision size, intraoperative blood loss, chest tube duration, and total drainage volume over the first three postoperative days. Complications were classified according to the Clavien-Dindo grading system. Pathological data included nodule diameter, histologic diagnosis, and margin status.

The long-term follow-up plan for patients diagnosed with lung cancer included the non-contrast CT scan of the chest, the non-contrast MRI cranial scan, the abdominal and superficial lymph nodes ultrasonography, and the serum pulmonary tumor marker test every three months after the surgery. The long-term follow-up plan for patients diagnosed with non-malignant lesions was non-contrast CT scan of the chest every six months after the surgery.

The long-term outcomes of our study are overall survival (OS) and recurrence-free survival (RFS). The OS rate was calculated from the date of surgery to the date of death, while the RFS rate was determined from the date of surgery to the date of disease recurrence. Patients who remained event-free at the last available follow-up date were right-censored at that date in the survival analysis.

### Statistical analysis

Propensity score matching was performed using logistic regression to estimate the probability of receiving CONSERVO or stapler-based resection, based on baseline characteristics (age, sex, smoking history, comorbidities, tumor size, and nodule location). Patients were matched 1:1 using the nearest neighbor method without replacement, with a caliper width of 0.2 standard deviations of the logit of the propensity score.

To account for potential confounding factors and evaluate the independent effects of surgical techniques and other variables on lung volume preservation, a multivariable linear regression analysis was performed. The dependent variable in the model was the percentage loss of lobe/lung volume, while the independent variables included age, sex, smoking history, comorbidities, nodule location, date of operation, surgical technique, incision length, and operative time. The regression model was used to estimate the adjusted effects of each independent variable while controlling the influence of the others. The assumptions of the linear regression model were assessed using residual-versus-fitted (RVF) plots for linearity and homoscedasticity, and variance inflation factors (VIFs) for multicollinearity. The RVF plots confirmed linearity and constant variance of residuals (**Figure 1**), while VIF values were all below 10, indicating no significant multicollinearity among the independent variables (**Supplementary Table S1**).

Continuous variables were tested by Shapiro-Wilk test to determine the type of distribution and were reported as mean ± standard deviation (SD) or median with interquartile range (IQR), and compared using the student’s t-test or Mann-Whitney U test, as appropriate. Categorical variables were analyzed using chi-square or Fisher’s exact test. A p-value < 0.05 was considered statistically significant. Statistical analysis was performed using STATA version 15.1 (StataCorp, TX).

## Results

### Patient characteristics

A total of 104 patients were included in the analysis: 49 in the stapler group and 55 in the CONSERVO group. Baseline characteristics, including age, sex, smoking history, comorbidities, prior malignancy, and nodule location, were well balanced between the groups. There were also no significant differences in tumor size or preoperative CT-based lung and nodule-residing lobe volumes. The was no baseline imbalance between the propensity score matched groups either (**Table 1**).

**Table 1:** Baseline characteristics of the patients.

Variable	The whole cohort			The propensity score matched cohort		
	Conventional n = 49	CONSERVO n = 55	p value	Conventional n = 33	CONSERVO n = 33	p value
Age (year)	61 (52–67)	52 (46–63)	0.060	55.06 ± 11.57	55.94 ± 12.50	0.768
Gender			0.206			0.602
Male	13 (26.53%)	21 (38.18%)		12 (36.36%)	10 (30.30%)	
Female	36 (73.47%)	34 (61.82%)		21 (36.64%)	23 (69.70%)	
Smoking (pack-year)	0 (0–0)	0 (0–0)	0.961	0 (0–0)	0 (0-0)	0.706
History of malignancy			0.056			1.000
Yes	7 (14.29%)	2 (3.64%)		33 (100%)	33 (100%)	
No	42 (85.71%)	53 (96.36%)		0 (%)	0 (%)	
Comorbidity			0.800			0.614
Yes	19 (38.78%)	20 (36.36%)		12 (36.36%)	14 (42.42%)	
No	30 (61.22%)	35 (63.64%)		21 (36.64%)	19 (57.58%)	
Location of the nodules			0.635			0.875
RUL	9 (18.37%)	16 (29.09%)		7 (21.21%)	7 (21.21%)	
RML	3 (6.12%)	4 (7.27%)		3 (9.09%)	4 (12.12%)	
RLL	14 (28.57%)	10 (18.18%)		8 (24.24%)	6 (18.18%)	
LUL	15 (30.61%)	17 (30.91%)		8 (24.24%)	11 (33.33%)	
LLL	8 (16.33%)	8 (14.55%)		7 (21.21%)	5 (15.15%)	
Preoperative lung volume (mm³)	2148286 (1785777-2482237)	2190096 (1891232-2675672)	0.542	2165607 (1917442-2378305)	1992677 (1706236-2514809)	0.401
Preoperative lobe volume (mm³)	998464.2 ± 281831.1	992206.6 ± 334691	0.916	713650.1 ± 272186.8	780242.4 ± 294005	0.343

The normally distributed variable was presented as mean ± standard deviation, the non-normally distributed continuous variables were presented as median (IQR), the categorical variables were display as count(percentage%).

RUL, the right upper lobe; RML, the right middle lobe; RLL, the right lower lobe; LUL, the left upper lobe; LLL, the left lower lobe.

### Postoperative outcomes and pathological findings

None of the patients included have experienced postoperative complications. The CONSERVO group demonstrated a longer operative time compared to the stapler group (median and IQR, 133 minutes [103–170 minutes] vs. 62 minutes [51–104 minutes], p < 0.001), consistent with the technical demands of electrosurgical dissection and suturing. However, CONSERVO technique was associated with a significantly smaller incision length (median and IQR. 23 mm [20-25mm] vs. 35 mm [30-40mm], p < 0.001) and fewer intraoperative bleeding (median and IQR, 10 mL [10-20mL] vs. 20 mL [10-30mL], p = 0.020). No statistically significant differences were observed in chest tube duration or total drainage volume during the first three postoperative days. There were no intraoperative complications or conversions to anatomical resection in either group. In the propensity score matched groups, the differences of the operative time consumption (median and IQR, 133 minutes [103–162 minutes] vs. 64 minutes [51–91 minutes], p = 0.002) and the length of incision (median and IQR, 23 mm [23-25mm] vs. 35 [30-39mm], p <0.001) remained, but the difference of intraoperative bleeding (median and IQR, 20 mL [10-20mL] vs. 20 mL [10-27mL], p=0.136) did not.

All the operation achieved R0 resection. None of the patients experienced local or distant recurrence up to the time of the data collection of the study. The distribution of pathological diagnoses was comparable between groups. Rates of adenocarcinoma *in situ* (AIS), minimally invasive adenocarcinoma (MIA), invasive adenocarcinoma, and benign lesions did not differ significantly. Furthermore, the size of the resected nodule was not significantly different. For the propensity score matched groups, similar pathological results remained (**Table 2**). The Median follow-up time was 15 months (6 to 30 months). No recurrence or death event occurred yet, so further survival analysis was not conducted.

**Table 2:** Perioperative outcomes of the patients.

Variable	The whole cohort			The propensity score matched cohort		
	Conventional n = 49	CONSERVO n = 55	p value	Conventional n = 33	CONSERVO n = 33	p value
Incision length (mm)	35 (30-40)	23 (20-25)	0.000	35 (30-39)	23 (23-25)	0.000
Operative time (min)	62 (51-104)	133 (103-170)	0.000	64 (51-91)	133 (103-162)	0.002
Operative bleeding volume (mL)	20 (10-30)	10 (10-20)	0.020	20 (10-27)	20 (10-20)	0.136
Time of drainage tube placement (days)	2 (2-3)	3 (2-3)	0.182	2 (2-3)	3 (2-3)	0.352
Drainage volume within 3 postoperative days (mL)	125 (60-235)	110 (55-170)	0.147	125 (75-205)	115 (60-172)	0.473
Diameters of the Nodule	7 (6-10)	7 (5-10)	0.687	8.42 ± 2.86	8.45 ± 3.36	0.969
Pathological results			0.993			0.897
Benign Lesions	3 (6.12%)	4 (7.27%)		3 (9.09%)	4 (12.12%)	
Adenocarcinoma In Situ	12 (24.49%)	13 (23.64%)		4 (12.12%)	4 (12.12%)	
Minimally Invasive Adenocarcinoma	17 (36.49%)	17 (30.91%)		14 (42.42%)	12 (36.36%)	
Invasive Adenocarcinoma	15 (30.61%)	19 (34.55%)		10 (30.30%)	13 (39.39%)	
Squamous Cell Carcinoma	1 (2.04%)	1 (1.82%)		1 (3.03%)	0 (0%)	
Secondary Carcinoma	1 (2.04%)	1 (1.82%)		1 (3.03%)	0 (0%)	

The non-normally distributed continuous variables were presented as median (IQR), the categorical variables were displayed as count(percentage%).

Lastly, due to surgeon heterogenicity, we performed a linear regression analysis on the operative time consumption by one surgeon XN. During this study, XN performed 33 CONSERVO resections, and the linear regression suggested a coefficient of -0.043833, i.e., approximately 13.14 minutes operative time consumption reduction per month, with 95% CI of -0.1030986 to 0.0154326, p=0.142 (**Supplementary Figure S2**; **Supplementary Table S2**).

### Parenchymal volume preservation

The CONSERVO group exhibited substantially less parenchymal loss, i.e., lobe/lung volume ratio loss (median and IQR, 4.66% [1.68–7.02%] vs. 11.17% [8.91–14.48%], p < 0.001) and lobe volume loss (median and IQR, 150057.2mm^3^ [63718.09-258075.2mm^3^] vs. 332481.7mm^3^ [209441-388930.2mm^3^], p<0.001). However, the total lung volume loss (mean and SD, 149855.5 ± 221949.3 mm³ vs. 220374.6 ± 189597.7 mm³, p = 0.086) only showed a non-significant trend favoring CONSERVO technique. In the PSM analysis, the lung volume-preserving effects of the CONSERVO technique compared to stapler-based resection remained statistically significant: lobe/lung volume ratio loss (mean and SD, 4.34 ± 4.09% vs. 10.89 ± 4.91%, p<0.001) and lobe volume loss (mean and SD, 155136.7 ± 122093.5mm^3^ vs. 320669.3 ± 130814mm^3^, p<0.001) (**Table 3**).

**Table 3:** Volumetric outcomes of the patients.

Variable	The whole cohort			The propensity score matched cohort		
	Conventional n = 49	CONSERVO n = 55	p value	Conventional n = 33	CONSERVO n = 33	p value
Lung Volume reduction (mm³)	220374.6 ± 189597.7	149855.5 ± 221949.3	0.086	194931.5 ± 145192.1	147563.1 ± 205539	0.284
Percentage of Lung Volume reduction (%)	10.34 ± 9.82	6.69 ± 10.10	0.066	8.48 ± 6.15	6.81 ± 9.91	0.415
Lobe volume reduction (mm³)	332481.7 (209441-388930.2)	150057.2 (63718.09-258075.2)	0.000	320669.3 ± 130814	155136.7 ± 122093.5	0.000
Percentage of Lobe Volume reduction (%)	33.79 ± 14.05	16.98 ± 13.20	0.000	31.90 ± 11.50	16.11 ± 13.30	0.000
Lobe/Lung volume ratio reduction (%)	11.17 (8.91-14.48)	4.66 (1.68-7.02)	0.000	10.89 ± 4.91	4.34 ± 4.09	0.000

The normally distributed variables were presented as mean ± standard deviation, the non-normally distributed continuous variables were presented as median (IQR).

The multivariable linear regression analysis identified three significant predictors of the percentage loss of lobe/lung volume: surgical technique, incision length, and operative time consumption. Patients who underwent CONSERVO resection experienced a significantly lower percentage loss of lobe/lung volume ratio compared to those who underwent stapler-based resection, with an average reduction of 13.3% (Coef. = -0.133, 95% CI: -0.182 to -0.084, p < 0.001). Incision length was inversely associated with percentage loss of lobe/lung volume ratio (Coef. = -0.00295, 95% CI: -0.00576 to -0.00014, p = 0.04), indicating that longer incisions were correlated with smaller reductions in lobe/lung volume ratio. Additionally, operative time was positively associated with lobe/lung volume ratio loss (Coef. = 0.00042, 95% CI: 0.00014 to 0.00071, p = 0.004), with each additional minute of operative time corresponding to a 0.042% increase in percentage loss of the lobe/lung volume ratio. No significant associations with the lobe/lung volume ratio were observed for other variables, including age, sex, smoking history, comorbidities, nodule location, or date of operation (p > 0.05) (**Table 4**).

**Table 4:** Results of the multivariable linear regression analysis on the lobe/lung volume ratio loss.

Independent variables	Coefficient	Std. Err.	t	P>t	[95% Conf. Interval]	
Age	0.0005048	0.0005015	1.01	0.317	-0.0004911	0.0015006
Gender	-0.0168743	0.015217	-1.11	0.270	-0.047088	0.0133394
Smoking History	-0.0015909	0.001506	-1.06	0.294	-0.0045811	0.0013993
Comorbidity	0.0270207	0.0144041	1.88	0.064	-0.0015791	0.0556204
Location of the nodule	0.0038817	0.0047243	0.82	0.413	-0.0054985	0.0132618
Date of the operation	-0.0000161	0.0000429	-0.37	0.709	-0.0001014	0.0000692
Resection method	-0.133053	0.0247009	-5.39	**0.000**	-0.1820973	-0.0840088
Incision Length	-0.0029521	0.0014142	-2.09	**0.040**	-0.00576	-0.0001441
Operative time consumption	0.0004234	0.0001427	2.97	**0.004**	0.00014	0.0007067
_cons	0.5236265	1.013589	0.52	0.607	-1.488878	2.536131

The table shows the effects of the independent variables on the dependent variable, i.e., the lobe/lung volume ratio loss. Noted that in the resection method variable, value 0 stands for the stapler-based resection and value 1 stands for CONSERVO resection.

The statistically significant values were presented in bold font.

## Discussion

The present study evaluated the CONSERVO technique—Conformal Sublobar ElectroResection with Volume Optimization—as an alternative to stapler-based wedge resection in the surgical management of small pulmonary nodules. Our findings demonstrate that the CONSERVO technique achieves significantly greater preservation of lung parenchyma while maintaining comparable safety, oncologic adequacy, and overall procedural effectiveness. Based on the result of the multivariable linear regression, we did not find significant factors associated reduced lobe/lung volume ratio loss other than the operation related ones, so theoretically, the patient who would benefit more from CONSERVO resection would be: 1) patients with limited pulmonary functional reserve; 2) patients who suffer from metachronous or synchronous high-risk nodules; and 3) patients with nodules that are located close to segmental or lobar structures. Additionally, the significantly shorter incision length suggests further cosmetic advantages and possibly a better quality of life, thus is more suitable for patients with cosmetic needs.

Regarding the metrics used to evaluate parenchymal preservation, we reported both absolute volume changes and volume ratios to ensure greater objectivity and interpretive clarity. Absolute lung and lobe volumes are known to fluctuate with the respiratory cycle and are particularly sensitive to the degree of inspiration achieved during CT acquisition. Forced inspiration, commonly employed during preoperative CT scans to achieve optimal image quality, may lead to overestimated lung volumes and increased variability across time points. Additionally, compensatory growth (8, 9) of parenchyma following resection can mask true tissue loss when evaluating whole-lung volumes alone. In contrast, the ratio of targeted lobe volume to ipsilateral lung volume more reliably reflects the relative impact of the resection. This ratio-based approach provides a more stable and physiologically grounded index of parenchymal preservation, mitigating variability from respiratory dynamics and compensatory inflation.

Compared to conventional stapler resection, the CONSERVO approach resulted in approximately a 50% reduction in lobe volume loss and a significant improvement in the lobe-to-lung volume loss ratio, this volume preserving effect was also suggested by the multivariable linear regression, which demonstrated a 13% reduction of the percentage loss of the lobe to the ipsilateral lung by the CONSERVO technique independent to other variables. These volumetric advantages are particularly meaningful in patients with limited pulmonary reserve or those likely to undergo repeat resections for synchronous and metachronous malignancies, of which the occurrence is fairly frequent (10). In another postoperative cohort, Shimada et al. reported that the nodule growth rate after the first resection in patients with multiple GGOs was 7.6% (11). Although the difference in total lung volume reduction between groups did not reach statistical significance, this observation lends additional support to our hypothesis: variability in forced inspiration during CT scanning and compensatory inflation of the remaining lung tissue can obscure true parenchymal loss when using absolute volumes alone. Considering the volumetric superiority of the CONSERVO approach, besides the contribution from the bilateral ventilation-based precise electrocautery dissection, another plausible contributor to the pronounced lobe volume preservation observed with the CONSERVO technique may be its capacity for lobule-based resection, which was enabled by precise electrocautery dissection under expanded, ventilated parenchyma in selected patients.

However, before hailing the triumph of this technique, one critical question must be addressed: Does preserving lung parenchyma reliably translate into functional protection? Obviously, techniques that conserve more than one lobe, such as bronchial sleeve resection, were both theoretically plausible and well-proven for their functional preservation capacity (12, 13). Nevertheless, the nuance and subtlety naturally increase when the scope dives into sub-lobar resection when the amount of parenchyma loss decreases (14), as it is well recognized that the relationship between anatomical preservation and postoperative lung function is influenced by multiple factors, including perfusion dynamics, ventilation distribution, and structural distortion after resection. For instance, large randomized controlled trials—most notably JCOG0802/WJOG4607L, which compared segmentectomy with lobectomy for early-stage non-small cell lung cancer—have shown only modest functional benefits of 3.5% in FEV1, not the predefined threshold of 10%, from segmentectomy (5) despite its theoretical advantage in parenchymal sparing. Similarly, smaller-scale retrospective comparisons between wedge resection and segmentectomy have yielded mixed results (4), reflecting the complex and often counterintuitive link between structure and function. These observations raise a critical issue: the mechanisms by which parenchymal preservation confers functional benefit remain incompletely understood.

Based on our findings, the answer may lie not solely in the quantity of preserved parenchyma but in its quality. Theoretically, under-ventilated parenchyma retained after non-anatomical resections may offer little functional value or, worse, contribute to air trapping or a ventilation-perfusion mismatch. Furthermore, deep or irregular resection planes can distort intersegmental architecture, compromising lobar mechanics and mucociliary clearance, which can affect normal ventilation. Additionally, poorly ventilated parenchyma would probably present as atelectatic tissue (15), hindering parenchyma re-expansion and recruitment, thus negatively impacting the lobe volume, as our study showed. However, evidence supporting the possible functional preservation effects of CONSERVO technique is currently lacking due to the retrospective nature of our study and objective limitations on the manpower of our study group. So the functional preserving effects of CONSERVO technique remain anatomically theoretical and hypothetical.

At the same time, it’s worth noting that the concept of individualized, precise resection of pulmonary nodules precedes the CONSERVO technique, and a multitude of centers have been conducting sub-segmentectomy or anatomical sub-lobar resection in accordance with the idea of conforming resection (16–18). However, it is cost-ineffective and unrealistic to attempt a more precise resection based on functional units smaller than sub-segments with the rigid and broad cutting line of stapler loads. Electrocautery resection in the setting of expanded coordinates brought by bilateral ventilation, on the other hand, could authentically follow the desired cutting plane with satisfactory accuracy. The substantial reduction in lobe-specific volume loss and the shape-preserving nature of the CONSERVO technique suggest that this technique may approach the anatomical precision of lobule-based resection in selected patients, provided careful preoperative preparation is employed. Although not formally recognized, we propose the term lobulectomy to describe this emerging surgical frontier: the selective resection of functional pulmonary lobules under direct vision and with preservation of broncho-vascular geometry. While speculative at this stage, the framework CONSERVO approach may offer new possibilities for treating sub-centimeter nodules in patients requiring maximal parenchymal preservation and warrants further anatomical and functional validation.

Despite these rationales, we emphasize that volume conservation does not automatically equate to functional preservation. Unfortunately, we did not have access to postoperative spirometry or perfusion imaging in this study. Hopefully, our volumetric data could provide a strong anatomical foundation upon which future functional studies can be built.

Oncological efficiency, the fundamental quality of any approach for nodule resection, is a significant consideration. For the CONSERVO approach, the negative resection margins and equivalent pathological profiles across both groups confirm that the pursuit of anatomical conformity does not compromise surgical thoroughness. As Ohtsuka et al. demonstrated, the electrocautery dissection of lung parenchyma is safe and feasible (19). Furthermore, electrocautery could theoretically sterilize the resection margin through thermal effects, as George et al. demonstrated in their ex vivo study, which showed electrocautery caused significant thermal damage compared to harmonic and conventional scalpel (20), thereby securing the margin while maintaining as much healthy parenchyma as possible. However, as it is well established that the disease-free survival of patients undergoing radial resection for early-stage NSCLC is relatively long, the long-term oncological effects of the CONSERVO approach still need to be examined in the future.

Interestingly, CONSERVO was associated with reduced intraoperative bleeding—a finding that may appear counterintuitive, given the expectation that stapler resections offer better hemostasis. However, the significance of the intergroups difference did not remain after propensity score matching. Unfortunately, given the sample loss during the propensity score matching, this change might result from the pseudo-randomization effect, or unsatisfactory statistical power. One pertinent hypothesis might explain this result. The strategically designed incision, namely the HilumDirect approach, which is currently under investigation in our center, of the CONSERVO approach not only allows for complex surgical maneuvers with a relatively short incision length, as shown in our results, but also has the potential to reduce the bleeding from incision and thoracic lymph node sampling.

Although the operative time was significantly longer with CONSERVO, this difference reflects the complexity and learning curve associated with fine anatomical dissection, rather than inherent procedural risk. In retrospect, the prolonged operative time did not result in adverse effects, such as an increase in the postoperative complication rate, prolonged thoracic drainage tube placement time, or increased postoperative drainage volume. Furthermore, for the most suitable set of patients who suffer from multiple metachronous nodules with high malignancy potential or limited functional reserve, a one-hour prolongation in operative time for a 13% reduction in lobe/lung volume ratio loss seems like fair trade. However, the multivariable linear regression model of our study suggested a positive association between operative time consumption and the lobe/lung volume loss, i.e., 0.042% more lobe/lung volume loss per minute for each operation. Although it’s theoretically plausible that the operative time might be a surrogate indicator of the operative complexity, thus positively correlated with more parenchyma loss, this finding strongly suggests the intricate relationship between surgical resection and parenchyma protection, thus warranting further randomized controlled trials to elucidate the effectiveness of CONSERVO technique.

We did not find a significant decrease in operative time over the period between 2023 and 2024 by linear regression analysis. One possible explanation is that multiple surgeons conducted surgeries in the CONSERVO cohort, and their learning capacities, which are especially important in technically demanding surgery, vary. Based on the linear regression on the operative time consumption of one surgeon, XN, who performed 33 CONSERVO resections during the study period, we found a none statistically significant reduction in operative time, i.e., approximately 13.14 minutes operative time consumption reduction per month, with 95% CI of -0.1030986 to 0.0154326, p=0.142, suggesting that merely 33 resections was probably not enough to stabilize and reduce the operative time consumption. However, there is a possible trend of further reduction of time consumption in the future. Another ongoing study is investigating the definitive study curve in our center.

We also found out that the incision length of CONSERVO resection was significantly shorter than that of the stapler-based resection. This finding reinforces the cosmetic gaining once the staplers were not required to conduct resection. For younger patients with cosmetic needs, the CONSERVO method provides a technique with smaller incision, and thus less obvious scar while maintaining the required resection margin. However, the multivariable regression suggested a small yet statistically significant reduction in the lobe/lung volume ratio loss while the length of the incision increased. This result reminds us that the over-emphasis on the control of incision length might compromise the parenchyma preservation, and resonates with the basic rationale of oncological surgery, i.e., cosmetic considerations come after the effective resection of the tumor and the preservation of physiological functions.

Theoretically speaking, the concept of the CONSERVO approach itself represents a departure from margin-guided resection toward a lobule-based, anatomy-respecting dissection. By leveraging lung expansion and utilizing electrocautery in the setting of the ventilated lobe to follow physiological planes, the CONSERVO approach enables tailored resections that more closely conform to the three-dimensional structure of the parenchyma. The ability to preserve interlobular and segmental geometry may also reduce postoperative deformation, potentially improving long-term respiratory mechanics—a hypothesis warranting further study. From a broader perspective, this work aligns with a movement in thoracic surgery: the shift toward function-preserving interventions without oncologic compromise. Segmentectomy has gained widespread adoption in this regard, but it is not suitable for every patient or lesion. In contrast, the CONSERVO approach offers a valuable niche for small, peripheral nodules that do not require anatomical segment resection but still benefit from a more thoughtful wedge approach.

This study has several limitations that should be acknowledged. First and foremost, its retrospective design inherently limits causal inference and is subject to selection bias, despite the well-balanced baseline characteristics. Although the patient demographics and clinical features between groups were comparable in both the original and further propensity score matching analysis generated groups, unmeasured confounders—such as surgeon experience, precise intraoperative judgment, and institutional preferences—may have influenced outcomes in subtle ways. Secondly, the absence of postoperative lung function testing (e.g., spirometry or perfusion scintigraphy) limits our ability to validate the functional significance of parenchymal preservation directly. While our volumetric measurements and ratio-based analysis provide an anatomical proxy, they do not fully capture dynamic physiological recovery, especially in patients with pre-existing lung disease or heterogeneous perfusion patterns. This limitation is especially relevant given the emerging understanding that function does not always correlate linearly with preserved volume. This absence of functional results of this retrospective study strongly suggests that the functional benefit from parenchyma preservation of CONSERVO technique remains theoretical until further clarification by functional tests. Hopefully we could shed some light on the functional preserving effects of the CONSERVO resection with the future follow-up on cardiopulmonary exercise testing for the patients and randomized controlled trial, before that, we must state clearly that the functional advantage of the CONSERVO resection should remain theoretical. Thirdly, our volumetric analysis was CT-based, which, while precise, is sensitive to variability in respiratory effort during scan acquisition. Although we used a stable and ratio-driven metric (i.e., lobe volume relative to ipsilateral lung volume), variations in inspiratory capacity, breath-hold technique, or scanner calibration might still contribute to measurement noise. Additionally, multiple surgeons were involved in the CONSERVO group, introducing inter-operator variability in technique execution and learning curves. This diversity might obscure or exaggerate operative time and intraoperative metrics. We attempted to mitigate this by standardizing key procedural steps and including only surgeons trained in the CONSERVO protocol; however, inconsistencies are unavoidable during the early adoption phases of a novel surgical strategy. Lastly, as the oncological efficiency of the resection being universally fundamental and irreplaceable to every new technique, the follow-up duration in this study is insufficient to assess long-term oncologic outcomes, such as recurrence-free or overall survival. Thus, the CONSERVO technique must be scrutinized with caution, as the long-term outcome of our study is immature yet. In all, although resection margins were uniformly negative and pathological results were comparable between groups, the true oncological durability of the CONSERVO approach must be confirmed in larger, longitudinal studies.

## Conclusion

In conclusion, the CONSERVO technique provides a safe, effective, and anatomically precise method for wedge resection. It enhances parenchymal preservation without compromising oncologic integrity and may represent an important step forward in function-oriented surgical management of pulmonary nodules. While the CONSERVO technique shows promise in preserving lung parenchyma and maintaining oncologic safety, this study should be viewed as hypothesis-generating. Future prospective, multicenter trials with functional and long-term oncologic endpoints are necessary to validate its broader applicability.

## Data Availability

The raw data supporting the conclusions of this article will be made available by the authors, without undue reservation.
