# Supplementary figures and images for: Conformal sublobar electroresection with volume optimization achieves greater parenchymal preservation than stapler in wedge resection: a volumetric analysis
Source: Front Oncol. 2025 Oct 27;15:1657405. doi: 10.3389/fonc.2025.1657405 (PMC12597738; doi:10.3389/fonc.2025.1657405)

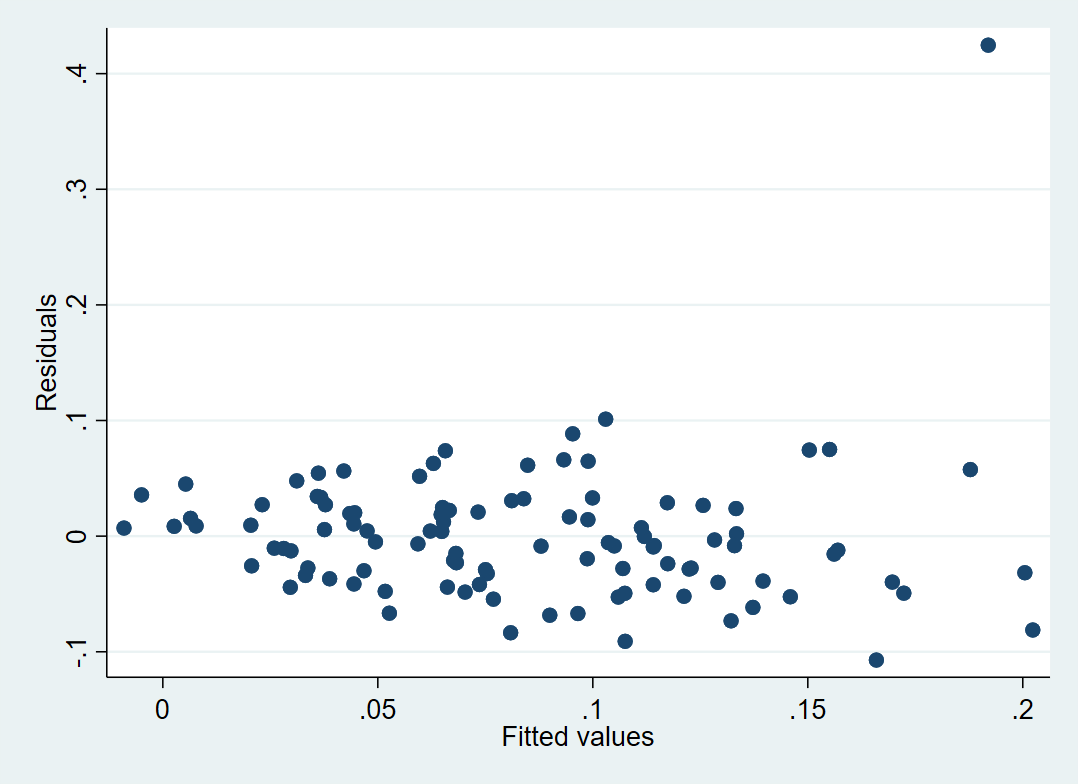

Supplement: Supplementary file 1 [file Image1.tif]

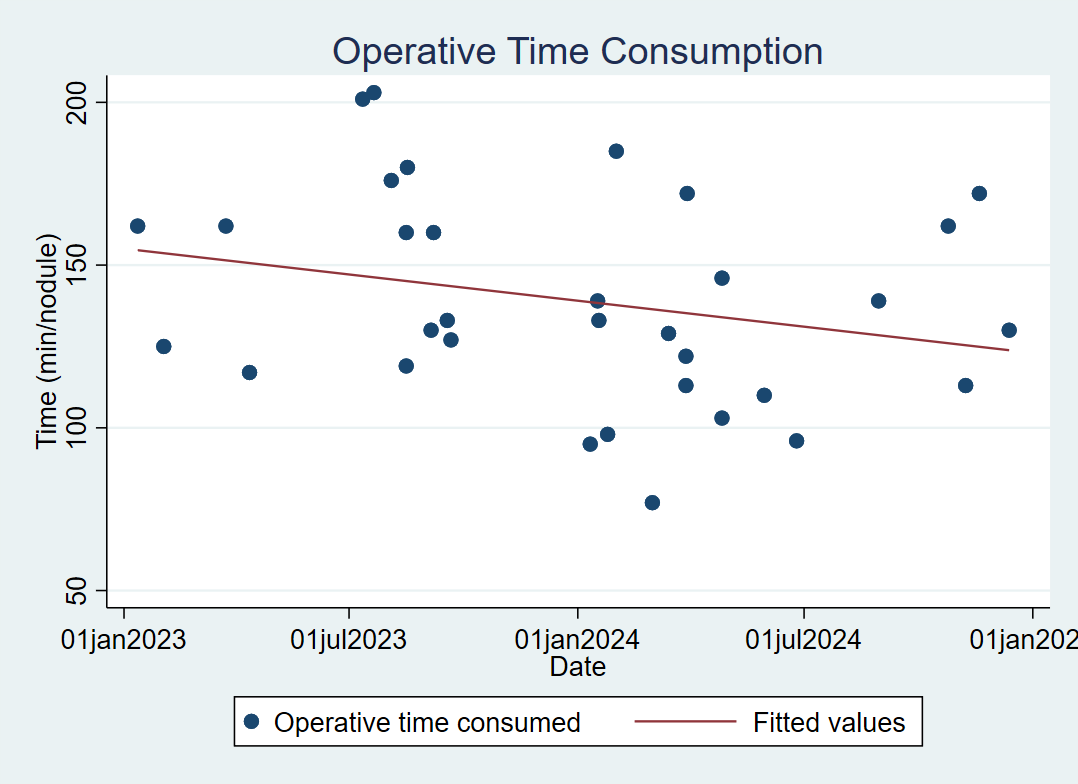

Supplement: Supplementary file 2 [file Image2.tif]
